# Type of adjuvant endocrine therapy and disease-free survival in patients with early HR-positive/HER2-positive BC: analysis from the phase III randomized ShortHER trial

**DOI:** 10.1038/s41523-023-00509-2

**Published:** 2023-02-04

**Authors:** Maria Vittoria Dieci, Giancarlo Bisagni, Stefania Bartolini, Antonio Frassoldati, Roberto Vicini, Sara Balduzzi, Roberto D’amico, Pierfranco Conte, Valentina Guarneri

**Affiliations:** 1 Department of Surgery, Oncology and Gastroenterology, University of Padova, via Giustiniani 2, 35128 Padova, Italy; 2 Oncology 2, Veneto Institute of Oncology IOV-IRCCS, via Gattamelata 64, 35128 Padova, Italy; 3 Department of Oncology and Advanced Technologies, Oncology Unit, Azienda USL-IRCCS, via Giovanni Amendola 2, 42122 Reggio Emilia, Italy; 4 Nervous System Medical Oncology Department, Istituto di Ricovero e Cura a Carattere Scientifico (IRCCS) Istituto delle Scienze Neurologiche di Bologna, Via Altura 3, 40139 Bologna, Italy; 5 Clinical Oncology, Department of Translational Medicine and for Romagna, S. Anna University Hospital, via Aldo Moro 8, 44124 Ferrara, Italy; 6 Department of Medical and Surgical Sciences for Children & Adults, University of Modena, via del Pozzo, 71, 41124 Modena, Italy; 7 Azienda Ospedaliero-Universitaria di Modena, Via del Pozzo, 71, 41124 Modena, Italy; 8 Veneto Oncology Network, via Gattamelata 64, 35128 Padova, Italy

**Keywords:** Breast cancer, Targeted therapies

## Abstract

The optimal adjuvant endocrine therapy for HR-positive/HER2-positive breast cancer patients is unknown. We included in this analysis 784 patients with HR-positive/HER2-positive BC from the randomized ShortHER trial of adjuvant trastuzumab (1 year vs 9 weeks) + chemotherapy. At a median follow-up of 8.7 years, patients who received AI had a significantly better DFS vs patients who received TAM or TAM-AI: 8-yr DFS 86.4 vs 79.7%, log-rank *P* = 0.013 (HR 1.52, 95% CI 1.09–2.11). In multivariate analysis, the type of endocrine therapy maintained a significant association with DFS (HR 1.64, 95% CI 1.07–2.52, *p* = 0.025 for TAM/TAM-AI vs AI). Among premenopausal patients aged ≤45 years, the use of GnRHa was associated with longer DFS: 8-yr DFS rate 85.2 vs 62.6% (log-rank *p* = 0.019, HR 0.41, 95% CI 0.19–0.88). In this post-hoc analysis of the ShortHER trial adjuvant treatment with AI was independently associated with improved DFS. Subgroup analysis in premenopausal patients suggests benefits with ovarian suppression.

**Trial registration:** NCI ClinicalTrials.gov number: NCT00629278.

## Introduction

Human epidermal growth factor receptor-2 (HER2)-positive breast cancer (BC) is a heterogeneous disease. The bidirectional cross-talk between the HER2 and the estrogen receptor pathways shapes biological differences in both molecular features and tumor microenvironment between hormone receptor (HR)-positive/HER2-positive and HR-negative/HER2-positive BC^1–3^. Clinical implications include different outcomes and treatment sensitivity^4^. For example, HR-positive/HER2-positive BC patients show a lower risk of relapse in the first 3–5 years after diagnosis as compared to patients with HR-negative/HER2-positive BC, however, the risk of relapse may persist longer at a later follow-up^5,6^. In the neoadjuvant setting, HR-positive/HER2-positive BC patients have a reduced chance of achieving a pathological complete response after neoadjuvant chemotherapy and anti-HER2 agents as compared to the HR-negative/HER2-positive subgroup^7,8^. Nevertheless, HR-positive/HER2-positive BC patients derive a similar degree of relative benefit from trastuzumab added to adjuvant chemotherapy^9,10^.

Endocrine therapy (ET) is a mainstay of treatment for HR-positive BC. In HR-positive/HER2-positive BC, the cross-talk between the two pathways may determine resistance to endocrine manipulation^11–14^. Although the addition of endocrine therapy to chemotherapy and anti-HER2-based neoadjuvant regimens does not improve the pathological complete response^15,16^, co-targeting the HER2 and the estrogen receptor pathway in HR-positive/HER2-positive BC is an effective strategy in the metastatic setting^17,18^ and the administration of adjuvant ET for 5–10 years in addition to chemotherapy and anti-HER2 treatment is standard in the adjuvant setting^19^.

Nevertheless, the optimal adjuvant ET for HR-positive/HER2-positive BC patients is still unclear. The 2015 EBCTCG metanalysis established aromatase inhibitors as the preferred treatment for postmenopausal patients with HR-positive BC based on a significant reduction in the risk of relapse of ~30% over tamoxifen^20^. This effect was maintained unchanged in both HER2-positive and HER2-negative BC subgroups^20^. However, HER2 status was not available for 70% of patients in this metanalysis and the HER2-positive subgroup was limited in sample size. Conversely, a combined analysis of 12,129 postmenopausal patients from three randomized trials of adjuvant ET with centralized HER2 evaluation demonstrated an interaction between type of adjuvant therapy (upfront tamoxifen or aromatase inhibitor) and HER2 status, with patients with HER2-negative disease deriving a greater benefit from aromatase inhibitor (HR 0.70, 95%CI 0.56-0.87) as compared to HER2-positive patients (HR = 1.13, 95% CI 0.75–1.71)^21^. In premenopausal patients with HR-positive BC undergoing ovarian function suppression (OFS), an aromatase inhibitor is superior to tamoxifen as demonstrated by the TEXT and SOFT trials, with a delta in distant disease-free survival (DFS) at 8 years of 4% (HR 0.77, 95% CI 0.67–0.90)^22^. However, HER2-negative BC patients derived benefit from aromatase inhibitor (5.4% absolute benefit in DFS at 8 years, HR 0.70), whereas tamoxifen was numerically superior in the HER2-positive subgroup (*n* = 695, 3.2% difference in DFS at 8 years, HR 1.18, 95% CI 0.80–1.73)^22^.

An important limitation of all these studies is the small proportion of HR-positive/HER2-positive patients who received anti-HER2 therapy as part of the systemic treatment. Therefore, there is a need to assess the optimal ET option for these patients in the context of standard adjuvant treatment including anti-HER2.

## Results

### Patients’ characteristics

We identified 853 patients with HR-positive/HER2-positive early BC in the ShortHER trial (68% of all randomized patients). Information on the type of adjuvant ET was available for 784 cases (92%). Patients’ characteristics according to the type of ET are shown in Table 1. More than half of patients (59.6%) received AI as adjuvant ET, 23.8% received TAM, and 16.6% TAM-AI. Patients receiving AI were older (*p* < 0.001) and more frequently in postmenopausal status (*p* < 0.001) as compared to patients treated with TAM or TAM-AI. There were no significant differences in the type of adjuvant ET according to disease stage, histologic grade, and randomization arm.

**Table 1:** Patients’ characteristics according to the type of adjuvant endocrine therapy.

		AI, *n* tot = 467		TAM, *n* tot = 187		TAM-AI, *n* = 130		Tot, *n* = 784		*p* value
		n	%	n	%	n	%	n	%	
**Age**	**Yrs, median (Q1; Q3)**	60 (55; 65)		43 (39;48)		49 (46;54)		55 (47; 63)		<0.001
**Menopausal status**	**Premenopause**	62	13,3%	162	86,6%	85	65,4%	309	39,5%	
	**Postmenopause**	404	86,7%	25	13,4%	45	34,6%	474	60,5%	<0.001
**Stage**	**I**	193	41,3%	73	39,0%	48	36,9%	314	40,1%	0.771
	**II**	199	42,6%	88	47,1%	61	46,9%	348	44,4%	
	**III**	75	16,1%	26	13,9%	21	16,2%	122	15,6%	
**Histologic Grade**	**1-2**	172	37,1%	64	35,0%	44	33,8%	280	36,1%	0.738
	**3**	291	62,9%	119	65,0%	86	66,2%	496	63,9%	
**Randomization arm**	**Long**	242	51,8%	92	49,2%	59	45,4%	393	50,1%	0.413
	**Short**	225	48,2%	95	50,8%	71	54,6%	391	49,9%	

*n* number, *tot* total, *yrs* years, *Q1* first quartile, *Q3* third quartile, *AI* aromatase inhibitor, *TAM* tamoxifen.

### Survival according to the type of adjuvant ET

At a median follow-up of 8.7 years (95% CI 8.6-8.8), 141 out of 784 patients had a DFS event (18.0%).

**Fig. 1:**
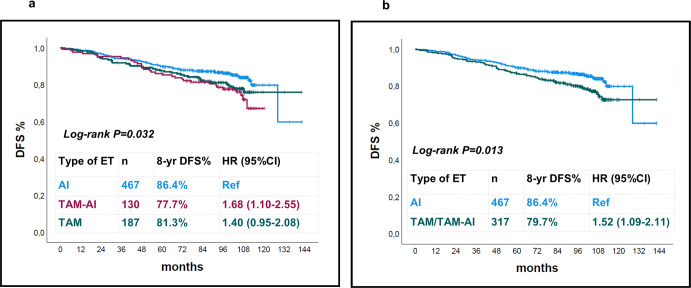
DFS Kaplan–Meier curves according to the type of adjuvant ET. Comparison of AI vs TAM-AI vs TAM (**a**) and comparison of AI vs TAM/TAM-AI (**b**).

DFS was significantly different according to the type of adjuvant ET received. At 8 years the DFS rates were: 86.4% for AI, 81.3% for TAM, and 77.7% for TAM-AI (log-rank *p* = 0.032; Fig. 1a). Univariate cox-regression analyses with the AI group as a reference showed an HR of 1.40 (95% CI 0.95–2.08, *p* = 0.089) for TAM and an HR of 1.68 (95% CI 1.10–2.55, *p* = 0.016) for TAM-AI. Since the AI group emerged as the one with the most favorable prognosis and patients treated with TAM or TAM-AI showed similar outcomes, we compared DFS for AI-treated vs TAM or TAM-AI-treated patients. DFS rates at 8 years were 86.4% for AI and 79.7% for TAM/TAM-AI, with an absolute difference of 6.7% (log-rank *p* = 0.013 Fig. 1b; HR = 1.52, 95% CI 1.09–2.11, *p* = 0.014). Table 2 shows the distribution of the type of DFS events per treatment group. The main effect of AI as compared to TAM or TAM-AI was in preventing locoregional or distant relapses.

**Table 2:** Summary of DFS events according to treatment group.

		AI *n* = 467		TAM *n* = 187		TAM-AI *n* = 130		Total *n* = 784	
		*n*	%	*n*	%	*n*	%	*n*	%
**DFS event (any)**		70	15.0%	39	20.9%	32	23.1%	141	18.0%
**DFS event type**	**Distant relapse**	34	7.3%	21	11.2%	15	11.5%	70	8.9%
	**Locoregional relapse**	8	1.7%	9	4.8%	8	6.2%	25	3.2%
	**Second primary (breast)**	7	1.5%	1	0.5%	1	0.8%	9	1.1%
	**Second primary (nonbreast)**	12	2.6%	7	3.7%	7	5.4%	26	3.3%
	**Death without prior event**	9	1.9%	1	0.5%	1	0.8%	11	1.4%

*DFS* disease-free survival, *AI* aromatase inhibitor, *TAM* tamoxifen, *n* number.

We conducted multivariate cox-regression analyses for DFS including the type of ET and other factors (Table 3). In model 1 we included those factors that were significantly associated with DFS in univariate analysis: type of ET, stage, and histologic grade. In model 2 we added menopausal status to factors included in model 1. We decided to include menopausal status since its potential confounding impact in the assessment of the effect of the type of adjuvant ET. In both models, adjuvant ET with TAM or TAM-AI was independently associated with worse DFS (HR 1.42, 95% CI 1.02–1.99, *p* = 0.040 in model 1; HR 1.64, 95% CI 1.07–2.52, *p* = 0.025 in model 2). For the representative purpose, separate DFS Kaplan–Meier curves of AI vs TAM/TAM-A in the subgroups of patients who were premenopausal or postmenopausal at study entry are reported as Supplementary Fig. 1. In terms of OS, there was no difference according to ET received (HR 0.89, 95% CI 0.54–1.49, *p* = 0.0667 for TAM/TAM-AI vs AI).

**Table 3:** Univariate and multivariate cox-regression models for DFS.

	Univariate		Multivariate model 1		Multivariate model 2	
	HR (95% CI)	*p*	HR (95% CI)	*p*	HR (95% CI)	*p*
**AI**	Ref		Ref		Ref	
**TAM or TAM-AI**	1.52 (1.09–2.11)	0.014	1.42 (1.02–1.99)	0.040	1.64 (1.07–2.52)	0.025
**Age (continuous)**	1.00 (0.98–1.02)	0.998				
**Stage I**	Ref		Ref		Ref	
**Stage II**	1.52 (1.02–2.26)	0.040	1.46 (0.98–2.18)	0.066	1.45 (0.97–2.17)	0.071
**Stage III**	2.82 (1.80–4.41)	0.001	2.79 (1.78–4.36)	<0.001	2.76 (1.77–4.32)	<0.001
**Histologic Grade 1-2**	Ref		Ref		Ref	
**Histologic Grade 3**	1.79 (1.22–2.63)	0.003	1.75 (1.19–2.58)	0.004	1.77 (1.21–2.61)	0.004
**Long arm Short arm**	Ref 1.08 (0.78–1.50)	0.655	-	-	-	-
**Postmenopausal status Premenopausal status**	Ref 1.17 (0.84–1.63)	0.369	-	-	Ref 0.80 (0.51–1.23)	0.303

*AI* aromatase inhibitor, *TAM* tamoxifen, *HR* hazard ratio, *CI* confidence interval, *p* *p* value, *Ref* reference.

**Fig. 2:**
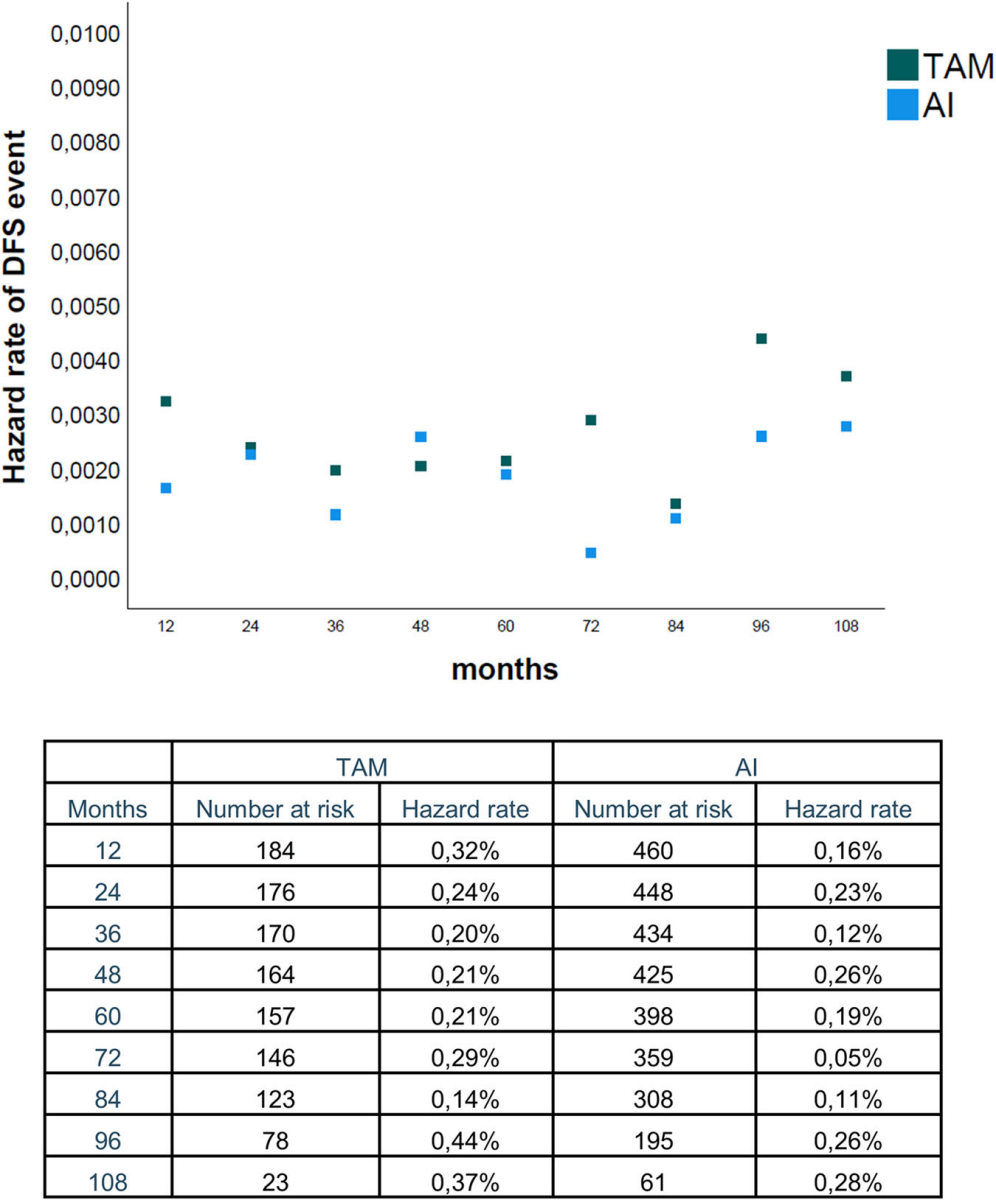
Annual hazard rates of DFS events for patients treated with AI or TAM. The last timepoint considered is 108 months since the very low number of patients at risk in later years.

We explored the annual hazard rates of DFS events in order to assess the benefit of AI over time. We excluded TAM-AI treatment from this analysis since this group included both patients switching from TAM to AI and the inverse sequence. As shown in Fig. 2, the annual hazard rate of DFS event for patients treated with AI were lower as compared to TAM at almost all timepoints considered, suggesting the benefit from AI on both early and late events. However, the duration of ET beyond 5 years was unknown, limiting the interpretation of these results.

### Premenopausal patients

We conducted exploratory analyses in the subgroup of 309 patients in premenopausal status at study entry based on the use of GnRHa as part of adjuvant ET. Table 4 summarizes patients’ characteristics according to GnRHa use.

**Table 4:** Patients’ characteristics according to GnRHa use in the premenopausal subgroup.

		GnRHa use: NO, *n* = 136		GnRHa use: YES, *n* = 173		Total		*P*
		*n*	%	*n*	%	*n*	%	
**Age**	**Years, median (Q1; Q3)**	49 (46; 51)		43 (39; 46)		46 (41; 49)		<0.001
**Stage**	**I**	61	44,9%	70	40,5%	131	42,4%	
	**II**	54	39,7%	78	45,1%	132	42,7%	
	**III**	21	15,4%	25	14,5%	46	14,9%	0.634
**Histologic Grade**	**1-2**	39	28,9%	60	35,5%	99	32,6%	
	**3**	96	71,1%	109	64,5%	205	67,4%	0.221
**Endocrine therapy**	**AI**	55	40,4%	7	4,0%	62	20,1%	
	**TAM or TAM-AI**	81	59,6%	166	96,0%	247	79,9%	<0.001
**Treatment arm**	**Long**	69	50,7%	83	48,0%	152	49,2%	
	**Short**	67	49,3%	90	52,0%	157	50,8%	0.630

*GnRH* gonadotropin-releasing hormone, *p* *p* value, *Q1* first quartile, *Q3* third quartile, *AI* aromatase inhibitor, *TAM* tamoxifen.

**Fig. 3:**
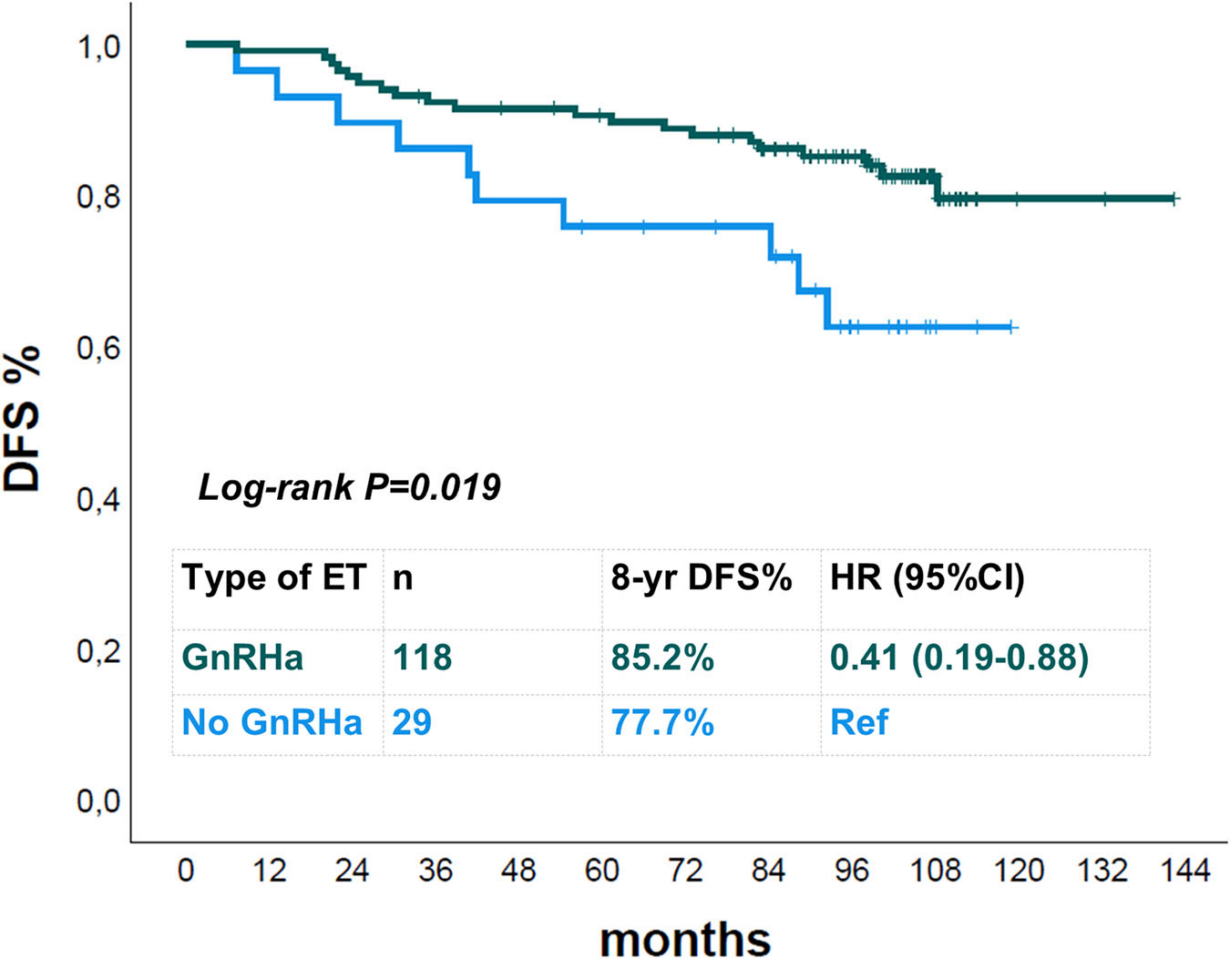
DFS Kaplan–Meier curves according to GnRHa use in premenopausal patients aged ≤45 years.

More than half of patients (56.0%, *n* = 173) received GnRHa as part of the adjuvant ET. These patients, as compared to those who did not undergo GnRH treatment, were significantly younger (*p* < 0.001). Almost all patients (96%) undergoing GnRHa received TAM or TAM-AI vs 59.6% not receiving GnRHa (*p* < 0.001). This apparently counterintuitive result was driven by 55 patients who received AI without GnRHa, likely on the basis of chemotherapy-induced amenorrhea. This hypothesis is supported by the median age of this subgroup of patients (premenopausal at study entry, no GnRHa use, treated with AI): 51 years, Q1:48; Q3: 53. In order to avoid potential confounding factors, we explored the impact of GnRHa on DFS by including only premenopausal patients aged ≤45 years (*n* = 147). In this group, 20% of patients did not receive GnRHa, 97% received TAM or TAM-AI, and only four patients received AI (combined with GnRHa). Thirty patients in this group experienced a DFS event. As shown in Fig. 3, GnRHa was associated with improved outcome: DFS rates at 8 years were 85.2 vs 62.6%, log-rank *p* = 0.019 (HR 0.41, 95% CI 0.19–0.88, *p* = 0.023).

## Discussion

In this work, we show that adjuvant ET with AI is superior to TAM or TAM-AI in terms of DFS for patients with HR-positive/HER2-positive BC receiving adjuvant anthracycline/taxane-based chemotherapy combined with trastuzumab. These results derive from an exploratory analysis of a randomized trial with 8.7 years of median follow-up. Conflicting results exist about a potential detrimental effect of HER2 overexpression on TAM efficacy^23–25^ and our data add to the ongoing debate about optimal adjuvant ET for patients with HR-positive/HER2-positive BC. The EBCTCG metanalysis did not demonstrate any interaction between HER2 status and benefit from AI over TAM in postmenopausal patients^20^. However, a recent trial-level meta-analysis addressing this clinical question provided contradictory findings. This study included 5390 HR-positive/HER2-positive BC patients (of whom 2410 in premenopausal status) from 6 randomized trials: five trials of adjuvant ET (TEAM, ATAC, BIG 1–98, TEXT, and SOFT) and one trial of adjuvant anti-HER2-therapy (ALTTO)^26^. The results showed no difference in DFS between adjuvant treatment with AI and TAM^26^. This meta-analysis has several limitations. The proportion of HR-positive/HER2-positive BC patients in most of these studies was small (in the range of 6 to 12%, excluding ALTTO) and HER2 status was not available for a large proportion of patients. Moreover, trastuzumab adjuvant treatment was administered to a minority of patients in the TEAM, ATAC, and BIG 1–98 trials, and only to 60% of HR-positive/HER2-positive BC patients from the TEXT and SOFT trials. In addition, the TEAM, ATAC, and BIG 1–98 results apply only to treatment and events occurring in the first 2–3 years of adjuvant ET.

Conversely, our analysis is based on a randomized trial dedicated to HER2-positive BC patients, including a large number of cases with HR-positive/HER2-positive BC (*n* = 784), all treated with standard adjuvant chemotherapy and trastuzumab. The type of ET was collected for the first 5 years of follow-up, median follow-up is long (8.7 years) and survival analysis refers to DFS events occurring throughout the follow-up period.

The superiority of AI demonstrated in our work is consistent with the results of a post-hoc analysis from the ALTTO trial. This analysis shows similarities with our work, since it included a large subgroup of HR-positive/HER2-positive BC patients (3603, of whom 1888 were premenopausal) from a clinical trial dedicated to HER2-positive BC patients all treated with adjuvant anti-HER2 therapy^6^. In multivariate analysis, AI was associated with better DFS as compared to TAM (HR 0.70, 95% CI 0.57–0.97)^6^. Conversely, also TAM-AI was associated with improved DFS as compared to TAM (HR 0.45, 95% CI 0.33–0.61)^6^. Differences in the classification of the type of ET might have impacted this discrepant finding. In our study, we define TAM-AI treatment when each drug was administered for at least 1 year. No information on the methods applied to categorize TAM-AI treatment is available for the ALTTO analysis. It should also be noted that the TAM-AI group in our study, although including patients who received at least 1 year of each drug, is highly heterogeneous in terms of timing of drugs (TAM or AI first), overall duration (not fully captured in our database) and duration of each single agent. Another difference between our study and the analysis conducted in the ALTTO trial is in the proportion of postmenopausal patients, slightly higher in our cohort (60.7 vs 52.4% in ALTTO). Moreover, the use of OFS in the ALTTO trial was very limited (<1% of the study population).

Cumulative evidence suggests that the benefit from AI over TAM in HR-positive/HER2-positive BC patients may be dependent on menopausal status. Although we show an association with improved DFS for the use of AI over TAM/TAM-AI that is independent from menopausal status in multivariate analysis, our data should be considered more informative for postmenopausal or perimenopausal patients and less informative for true premenopausal patients. Our population includes a large proportion of postmenopausal patients and, among those defined as premenopausal at study entry, there were probably patients in perimenopausal status who achieved effective OFS following chemotherapy. In addition, only a few premenopausal patients aged ≤45 years received AI, therefore conclusions about the optimal adjuvant ET for premenopausal patients can not be drawn based on our results.

In the TEXT and SOFT trials, for patients undergoing OFS, TAM was numerically superior to AI in the subgroup of HER2-positive BC patients^22^. The recent EBCTCG metanalysis comparing AI vs TAM in the context of OFS for premenopausal patients also suggested greater benefit from AI vs TAM in HER2-negative disease than in HER2-positive disease (RR 0·65 vs 1·08, *p* = 0·021)^27^. However, the HER2-positive subgroup was limited in sample size with a small number of events. Moreover, the difference between HER2-positive and HER2-negative tumors did not reach statistical significance^27^. A population-based cohort study from the Netherlands Cancer Registry including 1155 HR-positive/HER2-positive BC patients reported a significant benefit from AI over TAM in perimenopausal patients (age <45 to ≤55 years as a proxy), a numerical benefit in postmenopausal patients (age >55 years), and a lack of benefit for premenopausal patients (age ≤45 years)^28^. Limitations of this analysis are the heterogeneous administration of adjuvant trastuzumab, the heterogeneous use of OFS for premenopausal patients, and the lack of a clinical definition of menopausal status. However, these findings further support the hypothesis that hormonal microenvironment changes may affect the efficacy of ET in HR+/HER2 + BC patients.

A final key point of our work is the exploratory analysis of the role of OFS for premenopausal patients. The addition of GnRHa to ET (mainly TAM or TAM-AI) for premenopausal patients aged ≤45 years was associated with a significantly better DFS. Subgroup analyses of the SOFT trial demonstrated improved outcomes for OFS added to TAM in HER2-positive disease^22^. The Dutch population-based cohort study also described a survival benefit when OFS was added to ET for HR+/HER2 + BC patients^28^. In this work, we demonstrate the benefit of OFS in the context of a randomized trial including patients all treated with adjuvant chemotherapy and trastuzumab. The low rate GnRHa use in the ALTTO trial limits the possibility to explore this issue in that study^6^. It has to be noticed that the duration of GnRHa administration was not systematically collected. At the time the trial was conducted the optimal duration of OFS was unknown and OFS was generally administered for 2–5 years^29^.

Our study has limitations. First, this is an unplanned post-hoc analysis of a subgroup of 853 patients with a total of 141 DFS events. Second, it was not possible to properly assess therapy duration beyond 5 years and treatment adherence/interruptions. Moreover, perimenopausal patients were not clearly defined and other means of OFS beyond GnRHa were not captured after screening. It is true that all patients received trastuzumab, although half of them received 9-week treatment which is not the current standard. However, it is unlikely that the treatment duration might have biased our results, since the DFS multivariate analysis showing an independent role of ET type found no significant effect of the treatment arm. Finally, a large proportion of patients included in the ShortHER trial would be nowadays treated with a neoadjuvant approach followed by trastuzumab-emtansine in case of no pathological complete response, therefore the transferability of our data to contemporary real-world patients might not be straightforward.

In conclusion, our findings support the use of AI as adjuvant therapy for HR-positive/HER2-positive BC patients in the context of standard adjuvant treatment including chemotherapy and trastuzumab. In premenopausal patients, the use of OFS prolongs DFS. However, the optimal endocrine oral therapy (either AI or TAM) in this subgroup remains unclear and warrants further evaluation in large cohorts of patients treated with standard adjuvant therapy.

## Methods

### Study population and adjuvant ET

We included in this analysis patients with HR-positive (ER and/or PgR ≥10%) and HER2-positive BC enrolled in the ShortHER trial (NCT00629278) comparing 1 year vs 9-week trastuzumab added to anthracycline/taxane-based chemotherapy. Enrollment started in December 2007 and ended in October 2013. Study characteristics and results are reported elsewhere^30,31^. Adjuvant ET followed local standards according to guidelines. Options included:

- Tamoxifen (TAM), an aromatase inhibitor (AI), or TAM and AI in sequence for postmenopausal patients;
- TAM with or without OFS, TAM, and AI with or without OFS (switch to AI without OFS if postmenopausal status confirmed), AI with OFS for premenopausal patients and, in selected perimenopausal cases achieving amenorrhea following chemotherapy, AI without OFS with close monitoring of FSH, LH, and estradiol levels^32,33^.

The type of prescribed adjuvant ET was collected at each follow-up visit during the first 5 years from randomization and was classified as:

- aromatase inhibitor (AI),
- tamoxifen (TAM),
- tamoxifen and aromatase inhibitor (TAM-AI) in the case of both drugs were reported in at least two 6-month follow-up visits.

For patients in premenopausal status at study entry, OFS by gonadotropin-releasing hormone analogs (GnRHa) was also collected during follow-up.

### Statistical analysis

DFS was calculated from randomization to disease recurrence (locoregional or metastatic), second primary tumor, or death (any cause). OS was calculated from randomization to death.

Statistical analyses were performed using IBM SPSS v.24. Kaplan–Meier method was used to estimate survival curves. The log-rank test was used to compare between groups. Cox proportional regression models were used to calculate hazard ratios (HRs) and 95% confidence intervals (CIs). The significance level was *P* < 0.05. All tests were two-sided.

### Reporting summary

Further information on research design is available in the Nature Research Reporting Summary linked to this article.

## Supplementary information

Supplementary Information

Reporting Summary

## Data Availability

The datasets used and/or analysed during the current study are available from the corresponding author on reasonable request.
