# Author Correction: Type of adjuvant endocrine therapy and disease-free survival in patients with early HR-positive/HER2-positive BC: analysis from the phase III randomized ShortHER trial

**DOI:** 10.1038/s41523-023-00532-3

**Published:** 2023-04-12

**Authors:** Maria Vittoria Dieci, Giancarlo Bisagni, Stefania Bartolini, Antonio Frassoldati, Roberto Vicini, Sara Balduzzi, Roberto D’amico, Pierfranco Conte, Valentina Guarneri

**Affiliations:** 1 Department of Surgery, Oncology and Gastroenterology, University of Padova, via Giustiniani 2, 35128 Padova, Italy; 2 Oncology 2, Veneto Institute of Oncology IOV-IRCCS, via Gattamelata 64, 35128 Padova, Italy; 3 Department of Oncology and Advanced Technologies, Oncology Unit, Azienda USL-IRCCS, via Giovanni Amendola 2, 42122 Reggio Emilia, Italy; 4 Nervous System Medical Oncology Department, Istituto di Ricovero e Cura a Carattere Scientifico (IRCCS) Istituto delle Scienze Neurologiche di Bologna, Via Altura 3, 40139 Bologna, Italy; 5 Clinical Oncology, Department of Translational Medicine and for Romagna, S. Anna University Hospital, via Aldo Moro 8, 44124 Ferrara, Italy; 6 Department of Medical and Surgical Sciences for Children & Adults, University of Modena, via del Pozzo, 71, 41124 Modena, Italy; 7 Azienda Ospedaliero-Universitaria di Modena, Via del Pozzo, 71, 41124 Modena, Italy; 8 Veneto Oncology Network, via Gattamelata 64, 35128 Padova, Italy

**Keywords:** Breast cancer, Targeted therapies

Correction to: *npj Breast Cancer* 10.1038/s41523-023-00509-2, published online 04 February 2023

In this article the funding from ‘Veneto Institute of Oncology IOV-IRCCS “5x1000 anno di riferimento 2015—Genomica dei tumori e immunoterapia nell’era dei big data, fase 2” to VG’ was omitted. The original article has been corrected.

