# Supplementary material for: Type of adjuvant endocrine therapy and disease-free survival in patients with early HR-positive/HER2-positive BC: analysis from the phase III randomized ShortHER trial
Source: NPJ Breast Cancer. 2023 Feb 4;9:6. doi: 10.1038/s41523-023-00509-2 (PMC9899279; doi:10.1038/s41523-023-00509-2)
Supplement: Supplementary file 1 — Supplementary Information [file 41523_2023_509_MOESM1_ESM.pdf]

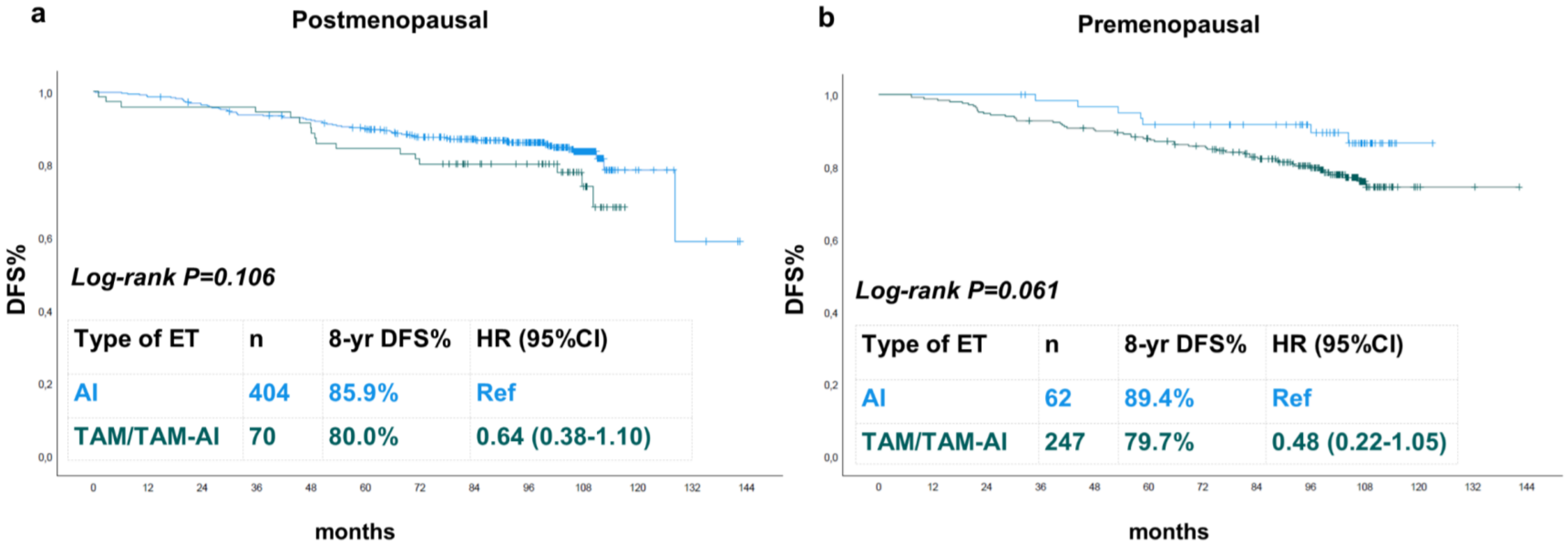

Supplementary Figure 1. DFS Kaplan Meier curves for AI vs TAM/TAM-AI in patients postmenopausal (a) or premenopausal (b) at study entry.
