# Supplementary material for: Type of adjuvant endocrine therapy and disease-free survival in patients with early HR-positive/HER2-positive BC: analysis from the phase III randomized ShortHER trial
Source: NPJ Breast Cancer. 2023 Feb 4;9:6. doi: 10.1038/s41523-023-00509-2 (PMC9899279; doi:10.1038/s41523-023-00509-2)
Supplement: Supplementary file 2 — Reporting Summary [file 41523_2023_509_MOESM2_ESM.pdf]

nature portfolio

Corresponding author(s): MARIA VITTORIA DIECI

Last updated by author(s): Jan 16, 2023

Reporting Summary

Nature Portfolio wishes to improve the reproducibility of the work that we publish. This form provides structure for consistency and transparency in reporting. For further information on Nature Portfolio policies, see our Editorial Policies and the Editorial Policy Checklist.

Statistics

For all statistical analyses, confirm that the following items are present in the figure legend, table legend, main text, or Methods section.

n/a	Confirmed	
☐	☒	The exact sample size (*n*) for each experimental group/condition, given as a discrete number and unit of measurement
☒	☐	A statement on whether measurements were taken from distinct samples or whether the same sample was measured repeatedly
☐	☒	The statistical test(s) used AND whether they are one- or two-sided<br>*Only common tests should be described solely by name; describe more complex techniques in the Methods section.*
☐	☒	A description of all covariates tested
☒	☐	A description of any assumptions or corrections, such as tests of normality and adjustment for multiple comparisons
☐	☒	A full description of the statistical parameters including central tendency (e.g. means) or other basic estimates (e.g. regression coefficient) AND variation (e.g. standard deviation) or associated estimates of uncertainty (e.g. confidence intervals)
☒	☐	For null hypothesis testing, the test statistic (e.g. *F*, *t*, *r*) with confidence intervals, effect sizes, degrees of freedom and *P* value noted<br>*Give P values as exact values whenever suitable.*
☒	☐	For Bayesian analysis, information on the choice of priors and Markov chain Monte Carlo settings
☒	☐	For hierarchical and complex designs, identification of the appropriate level for tests and full reporting of outcomes
☒	☐	Estimates of effect sizes (e.g. Cohen's *d*, Pearson's *r*), indicating how they were calculated

Our web collection on statistics for biologists contains articles on many of the points above.

Software and code

Policy information about availability of computer code

Data collection	https://servertrial.unimore.it/shorther/
Data analysis	IBM SPSS v.24

For manuscripts utilizing custom algorithms or software that are central to the research but not yet described in published literature, software must be made available to editors and reviewers. We strongly encourage code deposition in a community repository (e.g. GitHub). See the Nature Portfolio guidelines for submitting code & software for further information.

Data

Policy information about availability of data

All manuscripts must include a data availability statement. This statement should provide the following information, where applicable:

- Accession codes, unique identifiers, or web links for publicly available datasets
- A description of any restrictions on data availability
- For clinical datasets or third party data, please ensure that the statement adheres to our policy

The datasets used and/or analysed during the current study are available from the corresponding author on reasonable request.

Human research participants

Policy information about studies involving human research participants and Sex and Gender in Research.

Reporting on sex and gender	This study included patients with breast cancer, therefore unavoidably females.
Population characteristics	784 patients, all females. Median age 55 yrs (Q1:Q3 47:63), 39.5% premenopause, 40% stage I breast cancer., all HER2+ and hormone receptor (HR)+ breast cancer. All patients enrolled in the randomized shorther trial receiving adjuvant anthracycline-taxane based chemotherapy combined with trastuzumab for 9 weeks or 1 year.
Recruitment	Patients enrolled in a prospective randomized phase III trial. We focus in this paper on patients with hormone receptor-positive breast cancer for whom information ona djuvant endocrine therapy was available. Data on adjuvant endocrine therapy wass not available for only 8% of hormone receptor-positive breast cancer patients, so bias selection is unlikely.
Ethics oversight	The ethical committes of all participating centers approved the protocol, which was also approved by the Agenzia Italiana del Farmaco AIFA.

Note that full information on the approval of the study protocol must also be provided in the manuscript.

Field-specific reporting

Please select the one below that is the best fit for your research. If you are not sure, read the appropriate sections before making your selection.

☒ Life sciences ☐ Behavioural & social sciences ☐ Ecological, evolutionary & environmental sciences

For a reference copy of the document with all sections, see nature.com/documents/nr-reporting-summary-flat.pdf

Life sciences study design

All studies must disclose on these points even when the disclosure is negative.

Sample size	atients enrolled in a prospective randomized phase III trial. We focus in this paper on patients with hormone receptor-positive breast cancer for whom information ona djuvant endocrine therapy was available. Total of patients included 784: this is one of the alrgerst cohort of HER2+/HR+ BC patients analyzed for the type of adjuvant endocrine therapy, all homogeneously treated with adjuvant anthracycline and taxane-based chmeotherapy and trastuzumab.
Data exclusions	No data were excluded
Replication	not applicable
Randomization	Patients randomized to two different durations of trastuzumab. No randomization for the type of adjuvant endocrine therapy, which is the focus of this analysis. We have stated the exploratory nature of our work.
Blinding	Blinding not possible, we have stated the limits of our work.

Reporting for specific materials, systems and methods

We require information from authors about some types of materials, experimental systems and methods used in many studies. Here, indicate whether each material, system or method listed is relevant to your study. If you are not sure if a list item applies to your research, read the appropriate section before selecting a response.

Materials & experimental systems

n/a	Involved in the study	
☒	☐	Antibodies
☒	☐	Eukaryotic cell lines
☒	☐	Palaeontology and archaeology
☒	☐	Animals and other organisms
☐	☒	Clinical data
☒	☐	Dual use research of concern

Methods

n/a	Involved in the study	
☒	☐	ChIP-seq
☒	☐	Flow cytometry
☒	☐	MRI-based neuroimaging

March 2021

Clinical data

Policy information about clinical studies

All manuscripts should comply with the ICMJE guidelines for publication of clinical research and a completed CONSORT checklist must be included with all submissions.

Clinical trial registration	NCI ClinicalTrials.gov number: NCT00629278
Study protocol	The main publication with the results of the primary endpoint of the shorther trial have already been published (https://doi.org/10.1093/annonc/mdy414). Protocol availability was not required by the editorial office for the main publication.
Data collection	Enrollment started in December 2007 and ended in October 2013. Data collection for survival update is still ongoing.
Outcomes	In this analysis we adopted the primary and secondary survival endpoint of the Shorther-trial. DFS was calculated from randomization to disease recurrence (locoregional or metastatic), second primary tumor, or death (any cause). OS was calculated from randomization to death.
